# Use of ambient AI scribe in physicians’ clinical documentation: a protocol for a systematic review on effectiveness, efficiency, and satisfaction

**DOI:** 10.1136/bmjopen-2025-115562

**Published:** 2026-04-02

**Authors:** Carolina Garcia Sanchez, Vera Goer, Anna Kharko, Maria Hägglund, Josefin Hagström, Julian Schwarz, Charlotte R Blease

**Affiliations:** 1 Department of Women's and Children’s Health, Uppsala University, Uppsala, Sweden; 2 Department of Psychiatry and Psychotherapy, Immanuel Hospital Rüdersdorf Brandenburg, Medical School Theodor Fontane, Rüdersdorf bei Berlin, Germany; 3 Faculty of Health Sciences Brandenburg, Brandenburg Medical School Theodor Fontane, Neuruppin, Germany; 4 Centre for Primary Care and Health Services Research, Faculty of Biology, Medicine and Health, University of Manchester, Manchester, UK; 5 Uppsala University Hospital, Uppsala, Sweden; 6 Digital Psychiatry, Department of Psychiatry, Beth Israel Deaconess Medical Center, Harvard Medical School, Boston, Massachusetts, USA

**Keywords:** Health informatics, Artificial Intelligence, Electronic Health Records, Physicians, Systematic Review

## Abstract

**Abstract:**

**Introduction:**

Clinical documentation is a significant driver of burnout among physicians. Ambient artificial intelligence (AI) scribes, which leverage generative large language models to automate the creation of clinical notes from patient–physician conversations, are rapidly emerging as a potential solution. While these tools promise to enhance efficiency and reduce administrative tasks, concerns about the quality, accuracy and potential biases persist. There is now a need for a systematic synthesis of evidence to evaluate the impact of these technologies in clinical practice. To assess the effects of ambient AI scribes on physicians’ clinical documentation, the specific objectives are to: (1) evaluate the effectiveness of these tools on documentation, including accuracy and completeness; (2) synthesise evidence on the impact on physician efficiency after adoption, including time spent on documentation and (3) examine physicians’ satisfaction with these tools, including physicians’ perceived burden.

**Methods and analysis:**

A systematic review of quantitative or mixed-method studies as well as preprints will be conducted. We will perform a comprehensive search of four electronic databases (PubMed, IEEE Xplore, APA PsycInfo and Web of Science, along with medRix and ClinicalTrials.gov for preprints) for empirical studies published between January 2023 and March 2026. The review will synthesise studies comparing physicians’ use of ambient AI scribes with traditional documentation approaches. Given the anticipated heterogeneity of the studies, a narrative synthesis will be employed to summarise the findings. Where common quantitative outcomes exist, effect sizes will be calculated using Hedges’ g, mean differences or risk ratios/odds ratios as appropriate. The overall quality of evidence will be assessed using the Grading of Recommendations Assessment, Development and Evaluation (GRADE) framework.

**Ethics and dissemination:**

As no patient data are involved in the data collection, no ethical approval is acquired. Results will be disseminated in a peer-reviewed, open-access journal, and presented at relevant academic conferences.

**PROSPERO registration number:**

CRD420251149086.

**STRENGTHS AND LIMITATIONS OF THIS STUDY**

- This review’s methodology is a key strength, adhering to the Preferred Reporting Items for Systematic review and Meta-Analysis (PRISMA) protocol to ensure transparency and rigour.
- The inclusion of four languages broadens the scope of the evidence base beyond English-language literature.
- A primary limitation is the rapid evolution of artificial intelligence, which may quickly outdate the review’s findings.
- The exclusion of studies in other languages may introduce selection and publication bias.

## Introduction

### Background

 Clinical documentation and excessive administrative tasks are significant factors contributing to burnout among healthcare professionals (HCPs).^1^ Among HCPs, physicians play a central role in patient care and clinical decision-making.^2^ Administrative burden can compromise clinical tasks and also lead to reported impaired interactions between patient and practitioner^3^ and hence potentially the quality of care.^4^ Owing to these challenges, the integration of artificial intelligence (AI) for administrative healthcare tasks—particularly for documentation—has grown swiftly.^5^ Of particular relevance are generative artificial intelligence (genAI) technologies, a subset of AI which leverage large language models (LLMs) to learn patterns from vast data sets to generate novel content.^6^,^8^

In healthcare settings, the use of genAI tools for clinical documentation is emerging largely through ambient AI scribes which passively capture clinical conversations and automatically generate documentation in real time.^9^ Current commercial examples include Microsoft’s Nuance Dragon Ambient eXperience, ScribeAmerica’s Speke and Tandem Health. Physicians anticipate that AI could ease HCPs’ burden on clinical documentation.^10 11^ Preliminary studies suggest that chatbots and ambient AI scribes might streamline documentation processes, improve patient comprehension and increase the overall efficiency of healthcare services.^10^,^12^

However, the implementation of genAI tools in healthcare raises significant concerns^10 11 13^ about the overall quality of the generated documentation. For example, AI outputs can be biased, possibly perpetuating or even exacerbating ageism, racism or gender discrimination in healthcare due to omissions and algorithmic biases existing in the training data.^14^,^17^ Such biases can lead to unfair recommendations and discriminatory advice. Even in passive applications, such as ambient AI scribes, bias may arise in how speech is transcribed, summarised or prioritised—potentially misrepresenting dialects, accents or vernacular phrasing more commonly used by specific demographic groups.^18 19^ This could affect the completeness or accuracy of documentation and introduce inequalities into medical records.^20^ These tools may also generate unsafe medical advice and can produce inaccurate information called ‘hallucinations’.^17 21^ Users may be vulnerable to misinformation because of the convincing, rapid responses offered, which could further endanger privacy and safety.^22^ Further challenges arise in evaluating whether these tools perform better or worse than traditional physician-generated documentation, particularly in terms of accuracy, completeness and fairness.

### Rationale

Individual studies and pilot implementations^23^ have begun to explore the use of ambient AI scribe tools in clinical documentation. Similarly, some systematic reviews have been carried out to compare the use of AI-based tools for clinical documentation to traditional physician-generated documentation. Ng *et al*^24^ synthesised evidence on AI-driven transcription tools, including automatic speech recognition, LLMs and natural language processing systems, evaluating their accuracy, efficiency and usability in clinical practice. Their review encompassed a broad range of clinicians, such as physicians and nurses, and aimed to inform the integration of AI documentation technologies into clinical workflows.

Similarly, Bracken’s *et al*^25^ systematic review evaluated AI-driven documentation systems, including both genAI tools and ambient AI scribes, across various clinical settings and healthcare professions, such as physicians, nurses and allied health professionals. They found that these systems show considerable promise in enhancing documentation efficiency, with HCPs reporting ease of use and reduced workload. However, this review reported that issues of accuracy and consistency in AI-generated documentation remain.

Finally, Sessaville *et al*^26^ investigated the impact of AI scribes on clinicians, healthcare system efficiency, documentation processes and patient outcomes. This review included a heterogeneous set of healthcare providers across multiple clinical settings and examined a broad range of AI-scribe technologies, including ChatGPT-based tools and mobile applications. Although the included studies reported AI scribes have the potential to reduce documentation time and alleviate clinician burden, the breadth of technologies and professional roles limited role-specific conclusions. Part of their future research recommendations included that future reviews should consider stricter inclusion criteria.

The current evidence base remains scattered across various healthcare settings and disciplines, often treating HCPs as a homogeneous group without accounting for the distinct documentation requirements of different professional roles or genAI tools.^26 27^ This is particularly problematic given that clinical documentation is not only legally mandated but also differs substantially in form, content and purpose across medical specialties and professional groups. For example, physicians’ documentation serves crucial functions in guiding subsequent treatment and facilitating inter-professional communication, with requirements that may differ markedly from those of other HCPs (eg, Elkbuli *et al*^28^). To advance the field of genAI-assisted clinical documentation and measure its effects more rigorously, it is therefore essential to narrow the scope of investigation to specific professional contexts and documentation types and perform a more recent systematic review that includes the latest studies on this fast-evolving technology.

This review focuses particularly on ambient AI scribes because they represent a distinct type of genAI documentation tools that perform on a defined workflow step in comparison to, for example, the interactional nature of chatbots. These features create unique benefits and risks, including potential impacts on documentation accuracy, completeness, bias and workflow integration, that cannot be meaningfully evaluated when grouped with other, more heterogeneous genAI documentation tools, that is, traditional AI-based transcription systems, chatbots or standalone LLM tools. Prior reviews have combined multiple AI technologies and diverse clinical professions,^26^,^28^ which limit the specificity of their conclusions. Narrowing the focus to ambient AI scribes allows for a more rigorous and clinically relevant assessment of their effectiveness, efficiency and acceptability for physicians.

We therefore argue that a systematic review is urgently warranted–first, to synthesise current evidence on whether ambient AI scribes in healthcare deliver documentation quality, efficiency and usability that are comparable or superior to traditional physician-generated documentation; second, to limit it to physicians, who play a pivotal role in patient care, and third, to focus specifically on ambient AI scribes. To the best of our knowledge, no prior review has comprehensively addressed this critical gap.

This protocol details the methodology and approach that will guide the systematic review.

### Objectives

The aim of this review will be to systematically assess the impact of ambient AI scribes on physicians’ clinical documentation and documentation practices by synthesising evidence on objective and measurable outcomes.

To achieve this aim, the systematic review has the following objectives:

- To assess the impact of ambient AI scribes on the effectiveness, including accuracy, completeness and comprehensiveness of clinical documentation compared with traditional physician-generated documentation.
- To synthesise the evidence on ambient AI scribes’ impact on physicians’ efficiency and productivity, including time spent on documentation and effects on overall workflow and physicians’ productivity in comparison to customary documentation practices.
- To examine the evidence on satisfaction, including perceived burden of ambient AI scribes as a new tool for clinical documentation from the physician perspective.

## Methods and analysis

To address the objectives, we will conduct a systematic review of quantitative or mixed-method studies. Studies are expected to be highly heterogeneous in terms of design, target populations, variety of ambient AI scribes used and outcome measures as seen in previously conducted reviews.^24^,^26^ A scoping review was not selected, as the aim of this work is not to provide a broad mapping of the literature, but to critically assess and synthesise existing empirical evidence on specific uses of ambient AI scribes in physicians’ clinical documentation and documentation practices. Given the growing body of research in this domain, a focused and evaluative synthesis is both feasible and more suitable than an exploratory scoping review.

This protocol was created in accordance with the guidelines set forth by the Preferred Reporting Items for Systematic review and Meta-Analysis Protocols (PRISMA-P) and is documented following the PRISMA-P checklist (see online supplemental appendix 1).^29^ Research activities for this project commenced in June 2025, and the results are anticipated to be published by the end of 2026. Should any significant modifications to the protocol occur, the registration with PROSPERO will be updated accordingly. In such instances, a table detailing the changes along with their justifications will be included in the final review publication as is best practice.^30^

### Research questions

Based on the objectives, three research questions were formulated:

1. How do ambient AI scribes affect the quality and completeness of clinical documentation, in terms of accuracy, comprehensiveness and adherence to documentation standards compared with conventional physician-generated or customary documentation approaches? (EFFECTIVENESS)
2. What is the impact of ambient AI scribes on physician productivity, particularly regarding time spent on documentation and patient throughput, compared with conventional physician-generated or customary documentation approaches? (EFFICIENCY)
3. How do physicians perceive and rate the use of ambient AI scribes based on quantitative outcomes? (SATISFACTION)

### Eligibility criteria

#### Inclusion and exclusion criteria

The review question was elaborated using the Population, Exposure, Comparison and Outcome (PECO) statement, as outlined in table 1. Additionally, the inclusion and exclusion criteria are structured according to the PECO statement with additional elements incorporated as presented in more detail in table 1. For this review, we will consider studies from all healthcare settings, for example, public or private practice, hospitals and primary care clinics. The review will include empirical studies published between January 2023 and March 2026, including both peer-reviewed journal articles and preprints. Preprints are included to ensure that the review captures the most current empirical evidence in this rapidly evolving field. As preprints differ only in publication stage and not in study design, excluding them may overlook relevant findings on newly developed ambient AI scribe technologies. All preprints will be clearly marked as non-peer reviewed during data extraction. The selected timeframe also aligns with the surge in research following the public release of ChatGPT and the increased interest in genAI applications in healthcare. Given the rapid pace of technological innovation in this field, a broader timeframe would risk including studies based on vastly different technological foundations, which would confound a meaningful synthesis and comparison of the findings. Eligible studies must be published in English, Swedish, Spanish or German. The focus is on the use of LLM-based genAI models, specifically applied to clinical documentation processes in ambient AI scribes that support medical note-taking or record-keeping. Studies that exclusively investigate AI applications for diagnostic purposes without any relation to documentation will be excluded. Additionally, non-empirical literature such as reviews, opinion papers, editorials and theoretical works without original data will not be considered. Exceptions will be made for cases where, for example, a letter includes original data accompanied by sufficient methodological and analytical details.

**Table 1:** Population, Exposure, Comparison and Outcome (PECO) statement and eligibility criteria with additional details on setting, period and language

PECO	Description and inclusion criteria	Exclusion criteria
Population	Physicians using clinical documentation tools	Other HCP (eg, nurses, physiotherapists, dentists) or administrative staff
Exposure	Use of *ambient AI scribes* for the generation of all types of clinical documentation such as progress notes, discharge summaries, handover documents, clinic letters, operation notes	Use of AI technology only for clinical decision-making like diagnostics and other non-ambient genAI technologies, eg chatbots
Comparison	Comparisons between customary documentation methods (eg, manual entry into EHR, voice dictation without LLM-based genAI, etc) or alternative non-gen AI digital tools to ambient AI scribes	Comparisons between different generative AI-based documentation tools
Outcome	Objective/measurable outcome such as: Effectiveness of documentation (accuracy, completeness and comprehensiveness) Efficiency of documentation (time spent, effects on overall workflow and physician productivity) Satisfaction (including perceived burden)	Opinion, commentary, anecdotal reports or narrative descriptions without quantitative data or systematically collected outcome measures
Study design	All types of study design involving primary data collection and analysis (including quantitative mixed methods, preprints)	Non-empirical literature, reviews, opinion papers, editorials, theoretical works without original data
Setting	All medical disciplines, all healthcare settings, no location restrictions	Not medical discipline or healthcare setting
Period	Published between January 2023 and March 2026	Published before January 2023
Language	Published and available in English, Swedish, German and Spanish	Published in languages other than English, Swedish, German and Spanish

AI, artificial intelligence; EHR, electronic health record; HCP, health care professionals; LLM, large language models.

### Information sources

Searches will be conducted across six databases: PubMed, IEEE Xplore, APA PsycInfo and Web of Science along with medRix and ClinicalTrials.gov for preprints. Search terms will be adapted to fit the indexing and structure of each database.

### Search strategy

The PECO framework will guide the development of the search strategy, as detailed in table 1. The refinement of keywords will incorporate multiple approaches, including test searches performed by a librarian, review of references from relevant literature to identify commonly used terms and expert input from the research team. This approach helps to ensure that studies using varied terminology for the same/similar research questions will be captured.

The search strategy does not include the ‘Comparison’ element as this may limit search results. This modification maximises the retrieval of relevant data by ensuring no comparison constraints are applied in the search string. The ‘Comparison’ element will be retained during screening and synthesis to manage heterogeneity and preserve focus.

The preliminary search strategy for PubMed is provided in Multimedia online supplemental appendix 2.

### Study records

#### Data management

The literature search results will be imported into Rayyan (Rayyan Innovation, 2026), free web-based tool designed to assist on the screening and organise studies during systematic reviews. It allows for collaborative, blinded screening, tagging and filtering of articles based on inclusion and exclusion criteria, making the review process faster and more efficient.

#### Selection process

Following the completion of database searches, an experienced research librarian from Uppsala University, Sweden, will oversee the deduplication process to remove duplicate records from the search results. After deduplication, the screening process will be carried out. Reviewers will apply a decision tree and a detailed checklist, developed from the predefined inclusion and exclusion criteria, to independently assess titles, abstracts and full-text articles. Each record will be reviewed by at least two team members to ensure consistency. In cases of disagreement, the full text will be examined, and if necessary, a third reviewer will assist to resolve conflicts.

Full-text reports will be retrieved for all records that appear to meet the inclusion criteria or where there is any uncertainty. The first authors (CGS and VG) will then assess the full-text articles to determine their eligibility. Where necessary, additional information will be requested from study authors to clarify uncertainties. Disagreements or uncertainties will be resolved by discussion and, if needed, a third author will be included to make the final decision.

#### Data collection process

Data will be extracted independently by first authors (CGS and VG) using a standardised form. Discrepancies will be resolved through discussion or by a third author, and study authors will be contacted for clarification where necessary.

### Data items

#### Outcomes and prioritisation

Primary outcomes will be quantitative data on accuracy, comprehensiveness and completeness of the documentation (effectiveness), time spent on documentation as well as quantitative effects on the overall workflow and physicians’ productivity workflow (efficiency), and finally, the contentment and the perceived burden of the AI documentation (satisfaction). We expect this data to be expressed through a combination of validated psychometric scales and non-validated questionnaires, objective performance metrics and comparative analyses. The secondary outcome will be the differential effects observed between AI ambient scribe documentation and traditionally prepared reports, serving to identify the initial impacts of genAI tools on clinical workflows.

To address variability in how these outcomes may be reviewed, we will extract the specific measurement approach used in each study and assess its methodological rigour. Validated instruments will be noted when present but will not be required for inclusion. The expected heterogeneity in outcome definitions will be addressed through narrative synthesis.

#### Data synthesis

There is expected heterogeneity among the studies; however, if common quantitative outcomes emerge in the majority of included studies, we will calculate standardised mean differences, the Hedges’ g,^31^ to estimate effect sizes when studies use different measurement scales. If studies report outcomes using the same scale, mean differences will be calculated. For common categorical outcomes, effect sizes will be expressed as risk ratios or odds ratios, as appropriate. For other quantitative data, a qualitisation approach will be applied, transforming key themes into narrative summaries to complement the quantitative results and provide a comprehensive interpretation of the impact of ambient AI scribes.^32^

### Critical appraisal

#### Meta-bias and risk of bias in individual studies

Our review methodology incorporates a specific procedure to address the risk of systematic biases, including publication bias and selective outcome reporting. This involves cross-referencing the final published article with any available preregistered protocols or preprint manuscripts. We recognise that non-randomised studies are at a heightened risk for these biases, while simultaneously having a lower prevalence of preregistration. Consequently, the search strategy will extend to pre-print servers to broaden the evidence base and improve the overall transparency of the review.

#### Confidence in cumulative evidence

The Grading of Recommendations Assessment, Development and Evaluation (GRADE)^33^,^35^ approach will be used to assess the quality of evidence, providing a transparent evaluation of confidence in the cumulative evidence to guide clinical recommendations.

## Discussion

This review is designed to synthesise the current evidence on the impact of ambient AI scribes on physician’s clinical documentation. The findings will be critical for informing practitioners, policy-making and the future research agenda in this rapidly advancing field.

### Comparison with prior work

This systematic review aims to build on and refine the insights from previous research in the broader field of AI in healthcare documentation. Prior studies and reviews^2024^,^27 36^ have often taken a wider lens, examining various AI tools across a diverse population of HCPs. While valuable for mapping the landscape, these broader scopes can dilute the specific implications for any single professional group.

The key distinction of our work is its focused approach. By concentrating exclusively on physicians and the use of only ambient AI scribes, this review will provide a more targeted synthesis of evidence for a user group whose documentation carries immense legal and clinical weight. While earlier studies established that AI can reduce documentation burden for HCPs in general, 37,39our review aims to provide more specific evidence on ‘how’ and ‘to what extent’ ambient AI scribes impact physician-specific outcomes.

Furthermore, our defined timeframe (January 2023–March 2026) intentionally captures the large amount of research published in this field. This ensures that our findings will be more relevant to the current generation of technology than earlier reviews that may have assessed older, less capable systems. In essence, where prior work laid the foundation, this review will construct a more detailed and contemporary understanding, addressing a critical gap in the literature by focusing on the specific intersection of advanced AI scribes and the physician’s role in patient care.

### Strengths and limitations

The review protocol follows best-practice methodological rigour using the PRISMA protocol.^40^ Likewise, the review itself will follow the PRISMA guideline which is designed to improve the reporting of systematic reviews ensuring transparency and reproducibility.^41^ Likewise, the topic is highly relevant to clinical practice, with clear implications for the integration of AI tools in healthcare settings. Additionally, given the diversity of backgrounds within the team, sources not only in English but in Swedish, German and Spanish will also be included.

Still, as ambient AI scribes rapidly evolve, a potential limitation of the study is that the findings may soon become outdated. Furthermore, due to anticipated heterogeneity in study designs, outcome measures and AI tools used, the ability to synthesise findings—particularly through quantitative methods—could be limited. As a result, this may restrict generalisations associated with the findings. In addition, the exclusion of studies written in another language than English, Swedish, German or Spanish might lead to the omission of relevant studies. Therefore, our review may introduce a Western-centric bias, particularly given the global scale for potential generative AI adoption in healthcare. While resource limitations prevent full inclusion of non-English, non-Swedish, non-German and non-Spanish literature, studies with English-language abstracts will be screened when available to partially mitigate this limitation. The exclusion of grey literature, except for preprints, means that some studies will be overlooked. Nevertheless, our search strategy is designed to identify the most comprehensive and high-quality evidence. Finally, we have excluded patients’ experiences of ambient AI scribes. However, an understanding of patients’ perceptions is also critical and warrants dedicated focus in future research.

### Future direction

The findings of this systematic review will narrow critical gaps in the current body of literature, paving the way for a more targeted second generation of research. A primary future direction will be the development of standardised evaluation frameworks and reporting guidelines for studies on ambient AI scribes. The anticipated heterogeneity underscores the need for common metrics for effectiveness, efficiency and satisfaction to enable robust cross-study comparisons and future meta-analyses. Moreover, it is important to investigate the long-term effect of these tools for its clinical implementation. Finally, it is important that future research should be dedicated to algorithmic bias to not perpetuate existing health disparities.

### Ethics and dissemination plan

This study does not involve the collection or analysis of individual patient data; therefore, ethical approval is not required. To ensure that the findings of this systematic review reach relevant stakeholders and contribute meaningfully to practice, policy and future research, we plan to implement a comprehensive dissemination strategy. This will include publishing the results in a peer-reviewed, open-access journal and presenting them at relevant academic conferences and professional meetings.

## Supplementary material

10.1136/bmjopen-2025-115562 online supplemental file 1

10.1136/bmjopen-2025-115562 online supplemental file 2

## References

[1] Wu Y, Wu M, Wang C (2024). Evaluating the Prevalence of Burnout Among Health Care Professionals Related to Electronic Health Record Use: Systematic Review and Meta-Analysis. JMIR Med Inform.

[2] Adane K, Gizachew M, Kendie S (2019). The role of medical data in efficient patient care delivery: a review. Risk Manag Healthc Policy.

[3] Friedberg MW, Chen PG, Van Busum KR (2014). Factors Affecting Physician Professional Satisfaction and Their Implications for Patient Care, Health Systems, and Health Policy. Rand Health Q.

[4] Tierney AA, Gayre G, Hoberman B (2024). Ambient Artificial Intelligence Scribes to Alleviate the Burden of Clinical Documentation. NEJM Catalyst.

[5] Bongurala AR, Save D, Virmani A (2024). Transforming Health Care With Artificial Intelligence: Redefining Medical Documentation. *Mayo Clin Proc Digit Health*.

[6] Goodfellow IJ, Pouget-Abadie J, Mirza M, Ghahramani Z, Welling M, Cortes C (2014). Advances in neural information processing systems.

[7] Kingma DP, Welling M (2013). Auto-encoding variational bayes.

[8] Vaswani A, Shazeer N, Parmar N (2023). Attention is all you need, arxiv.

[9] Haberle T, Cleveland C, Snow GL (2024). The impact of nuance DAX ambient listening AI documentation: a cohort study. J Am Med Inform Assoc.

[10] Blease C (2024). Open AI meets open notes: surveillance capitalism, patient privacy and online record access. J Med Ethics.

[11] Blease C, Torous J (2023). ChatGPT and mental healthcare: balancing benefits with risks of harms. *BMJ Ment Health*.

[12] Goer V, Schwarz J KI-Dokumentation in der psychotherapie: qualitative studie zu nutzungserfahrungen von psychotherapeut:innen 2025.

[13] Schwarz J, Goer V, Guzhova O Psychiatrists’ Experiences and Opinions of Generative AI: An Exploratory Online Mixed Methods Survey in Germany.

[14] Ferrara E (2023). Should ChatGPT be biased? Challenges and risks of bias in large language models. *FM*.

[15] Gross N (2023). What ChatGPT Tells Us about Gender: A Cautionary Tale about Performativity and Gender Biases in AI. Soc Sci (Basel).

[16] King M (2022). Harmful biases in artificial intelligence. Lancet Psychiatry.

[17] Kotek H, Dockum R, Sun D (2023). Gender bias and stereotypes in large language models. https://dl.acm.org/doi/proceedings/10.1145/3582269.

[18] Martin JL, Wright KE (2023). Bias in Automatic Speech Recognition: The Case of African American Language. Applied Linguistics.

[19] Zolnoori M, Vergez S, Xu Z (2024). Decoding disparities: evaluating automatic speech recognition system performance in transcribing Black and White patient verbal communication with nurses in home healthcare. JAMIA Open.

[20] Eccles A, Pelly T, Pope C (2025). Unintended consequences of using ambient scribes in general practice.

[21] Ji Z, Lee N, Frieske R (2023). Survey of Hallucination in Natural Language Generation. ACM Comput Surv.

[22] Chen S, Kann BH, Foote MB (2023). Use of Artificial Intelligence Chatbots for Cancer Treatment Information. JAMA Oncol.

[23] Shah SJ, Devon-Sand A, Ma SP (2025). Ambient artificial intelligence scribes: physician burnout and perspectives on usability and documentation burden. J Am Med Inform Assoc.

[24] Ng JJW, Wang E, Zhou X (2025). Evaluating the performance of artificial intelligence-based speech recognition for clinical documentation: a systematic review. BMC Med Inform Decis Mak.

[25] Bracken A, Reilly C, Feeley A (2025). Artificial Intelligence (AI) - Powered Documentation Systems in Healthcare: A Systematic Review. J Med Syst.

[26] Sasseville M, Yousefi F, Ouellet S (2025). The Impact of AI Scribes on Streamlining Clinical Documentation: A Systematic Review. Healthcare (Basel).

[27] Perkins SW, Muste JC, Alam T (2024). Improving Clinical Documentation with Artificial Intelligence: A Systematic Review. Perspect Health Inf Manag.

[28] Elkbuli A, Godelman S, Miller A (2018). Improved clinical documentation leads to superior reportable outcomes: An accurate representation of patient’s clinical status. Int J Surg.

[29] Moher D, Stewart L, Shekelle P (2015). All in the Family: systematic reviews, rapid reviews, scoping reviews, realist reviews, and more. Syst Rev.

[30] Shamseer L, Moher D, Clarke M (2015). Preferred reporting items for systematic review and meta-analysis protocols (PRISMA-P) 2015: elaboration and explanation. BMJ.

[31] Hedges LV (1981). Distribution Theory for Glass’s Estimator of Effect Size and Related Estimators. J Educ Stat.

[32] Aromataris E, Lockwood C, Porritt K (2024). JBI manual for evidence synthesis.

[33] Prasad M (2024). Introduction to the GRADE tool for rating certainty in evidence and recommendations. Clin Epidemiol Glob Health.

[34] Atkins D, Eccles M, Flottorp S (2004). Systems for grading the quality of evidence and the strength of recommendations I: critical appraisal of existing approaches The GRADE Working Group. BMC Health Serv Res.

[35] Schünemann HJ, Brennan S, Akl EA (2023). The development methods of official GRADE articles and requirements for claiming the use of GRADE – A statement by the GRADE guidance group. J Clin Epidemiol.

[36] Ghatnekar S, Faletsky A, Nambudiri VE (2021). Digital scribe utility and barriers to implementation in clinical practice: a scoping review. Health Technol.

[37] Balloch J, Sridharan S, Oldham G (2024). Use of an ambient artificial intelligence tool to improve quality of clinical documentation. *Future Healthc J*.

[38] Barak-Corren Y, Wolf R, Rozenblum R (2024). Harnessing the Power of Generative AI for Clinical Summaries: Perspectives From Emergency Physicians. Ann Emerg Med.

[39] Owens LM, Wilda JJ, Hahn PY (2024). The association between use of ambient voice technology documentation during primary care patient encounters, documentation burden, and provider burnout. Fam Pract.

[40] Tricco AC, Lillie E, Zarin W (2018). PRISMA Extension for Scoping Reviews (PRISMA-ScR): Checklist and Explanation. Ann Intern Med.

[41] Page MJ, Moher D, Bossuyt PM (2021). PRISMA 2020 explanation and elaboration: updated guidance and exemplars for reporting systematic reviews.

