# Supplementary material for: Use of ambient AI scribe in physicians’ clinical documentation: a protocol for a systematic review on effectiveness, efficiency, and satisfaction
Source: BMJ Open. 2026 Apr 2;16(4):e115562. doi: 10.1136/bmjopen-2025-115562 (PMC13052704; doi:10.1136/bmjopen-2025-115562)
Supplement: online supplemental file 2 [file bmjopen-16-4-s002.docx]

**Appendix 2. Preliminary search strategy**

**PubMed**

| Search | Terms & fields |
| --- | --- |
| 1 | "Speech Recognition Software"[Mesh] OR "Natural Language Processing"[Mesh] OR "speech recognition software"[tiab] OR "natural language processing"[tiab] OR "ambient AI scribe*"[tiab] OR "ambient artificial intelligence"[tiab]  OR "ambient documentation"[tiab] OR "ambient clinical documentation"[tiab] OR "AI scribe*"[tiab] OR "automated documentation"[tiab] OR "automatic documentation"[tiab] OR  AI-assisted[tiab] |
| 2 | "Physicians"[Mesh] OR physician*[tiab] OR doctor*[tiab] OR clinician*[tiab] |
| 3 | "Documentation"[Mesh] OR "Medical records systems, computerized"[Mesh] OR "Electronic Health Records"[Mesh] OR "Health Records, Personal"[Mesh] OR "Patient Discharge"[Mesh] OR "Patient Discharge Summaries"[Mesh] OR documentation*[tiab] OR "medical record*"[tiab] OR "clinical note*"[tiab] OR "medical note*"[tiab] OR "clinical record*"[tiab] OR "patient discharge*"[tiab] OR "health record*"[tiab] OR "patient record*"[tiab] |
| 4 | 1 AND 2 AND 3 |
| 5 | (("Speech Recognition Software"[Mesh] OR "Natural Language Processing"[Mesh] OR "speech recognition software"[tiab] OR "natural language processing"[tiab] OR "ambient AI scribe*"[tiab] OR "ambient artificial intelligence"[tiab] OR "ambient documentation"[tiab] OR "ambient clinical documentation"[tiab] OR "AI scribe*"[tiab] OR "automated documentation"[tiab] OR "automatic documentation"[tiab] OR AI-assisted[tiab]) AND ("Physicians"[Mesh] OR physician*[tiab] OR doctor*[tiab] OR clinician*[tiab])) AND ("Documentation"[Mesh] OR "Medical records systems, computerized"[Mesh] OR "Electronic Health Records"[Mesh] OR "Health Records, Personal"[Mesh] OR "Patient Discharge"[Mesh] OR "Patient Discharge Summaries"[Mesh] OR documentation*[tiab] OR "medical record*"[tiab] OR "clinical note*"[tiab] OR "medical note*"[tiab] OR "clinical record*"[tiab] OR "patient discharge*"[tiab] OR "health record*"[tiab] OR "patient record*"[tiab]) |
