# Supplementary material for: Use of ambient AI scribe in physicians’ clinical documentation: a protocol for a systematic review on effectiveness, efficiency, and satisfaction
Source: BMJ Open. 2026 Apr 2;16(4):e115562. doi: 10.1136/bmjopen-2025-115562 (PMC13052704; doi:10.1136/bmjopen-2025-115562)
Supplement: Supplementary file 3 [file bmjopen-2025-115562.reviewer_comments.pdf]

PEER REVIEW HISTORY

BMJ Open publishes all reviews undertaken for accepted manuscripts. Reviewers are asked to complete a checklist review form and are provided with free text boxes to elaborate on their assessment. These free text comments are reproduced below.

ARTICLE DETAILS

Title (Provisional)

Use of Ambient AI Scribe in Physicians’ Clinical Documentation: A Protocol for a Systematic Review on Effectiveness, Efficiency and Satisfaction

Authors

Garcia Sanchez, Carolina; Goer, Vera; Kharko, Anna; Hägglund, Maria; Hagström , Josefin; Schwarz, Julian; Blease, Charlotte R

VERSION 1 - REVIEW

**Reviewer**	**1**
**Name**	**Crowe, Francesca**
**Affiliation**	**University of Birmingham, Institute of Applied Health Research**
**Date**	**04-Feb-2026**
**COI**	**None**

---

1. Could the authors provide a justification for the inclusion of only ambient AI scribes?

2. It would be useful to get an idea of some of the ways of measuring the outcomes proposed in this systematic review that will assess effectiveness of these AI scribes. How might the outcomes accuracy, completeness, and comprehensiveness be assessed if this is done so quantitatively. Will you accept any measure of these outcomes or should they have been measured using a validated instrument that captures the outcome adequately?

3. Are pre-prints a study design? Surely these would be either quantitative or mixed methods but they are not peer reviewed.

4. For deciding whether a article is eligible for inclusion, will the two team members be the same throughout this whole process? You have later stated that “pairs of reviewers will assess the full text articles”, how many pairs (i.e. how many separate people) will be included in this process?

5. If studies measure a similar outcome but use different scales could you calculate a standardised mean difference?

VERSION 1 - AUTHOR RESPONSE

Reviewer: 1
Dr. Francesca Crowe, University of Birmingham

Comments to the Author:
1. Could the authors provide a justification for the inclusion of only ambient AI scribes?
Thank you for this relevant and helpful comment. We have now added a justification for focusing exclusively on ambient AI scribes in the Rationale section, placed directly before the objectives.

2. It would be useful to get an idea of some of the ways of measuring the outcomes proposed in this systematic review that will assess effectiveness of these AI scribes. How might the outcomes accuracy, completeness, and comprehensiveness be assessed if this is done so quantitatively. Will you accept any measure of these outcomes or should they have been measured using a validated instrument that captures the outcome adequately?
Thank you for this helpful comment. We have now clarified in the Methods section under the "Outcomes and Prioritization" subsection how the outcomes related to documentation quality will be handled. Because accuracy, completeness, and comprehensiveness can be measured in different ways, we will include studies that use validated instruments where available, as well as studies that use clearly defined quantitative methods such as expert review, error counts, checklists, or guideline-based scoring. We will extract and report the exact measurement approach used in each study, assess methodological rigor, and synthesize results narratively to the expected heterogeneity.

3. Are pre-prints a study design? Surely these would be either quantitative or mixed methods but they are not peer reviewed.
Thank you for raising this point. This topic was discussed within the team, and we have chosen to include preprints because they differ only in publication status, not in study design. Preprints may report the same eligible quantitative or mixed-method designs as peer-reviewed articles but appear earlier in the publication process. Given the rapid development of ambient AI scribes, excluding preprints could result in missing very recent empirical evidence. We will consider their status in the interpretation of the findings. We have added more details about the reason for including preprints in the Eligibility criteria section in the manuscript.

4. For deciding whether a article is eligible for inclusion, will the two team members be the same throughout this whole process? You have later stated that "pairs of reviewers will assess the full text articles", how many pairs (i.e. how many separate people) will be included in this process?
Thank you for the question. All authors will take part in the title and abstract screening, with each record reviewed independently by two authors before moving to full-text assessment. Full-text screening will then be carried out primarily by the first authors, with senior authors being consulted to resolve any disagreements or uncertainties. We have clarified this process in the manuscript, specifically in the "Selection Process" section.

5. If studies measure a similar outcome but use different scales could you calculate a standardised mean difference?
Thank you for this comment. We have now clarified this process more explicitly in the manuscript.

Reviewer: 1
If you have selected 'Yes' above, please provide details of any competing interests.: N/A
We have reviewed the submission to ensure consistency, and we confirm that no competing interests are present.
